# In Vitro Imaging and Molecular Characterization of Ca^2+^ Flux Modulation by Nanosecond Pulsed Electric Fields

**DOI:** 10.3390/ijms242115616

**Published:** 2023-10-26

**Authors:** Francesca Camera, Eleonora Colantoni, Tomas Garcia-Sanchez, Barbara Benassi, Claudia Consales, Adeline Muscat, Leslie Vallet, Luis M. Mir, Franck Andre, Caterina Merla

**Affiliations:** 1 Division of Health Protection Technologies, Italian National Agency for Energy, New Technologies and Sustainable Economic Development (ENEA), 00123 Rome, Italy; francesca.camera@enea.it (F.C.); eleonora.colantoni@enea.it (E.C.); barbara.benassi@enea.it (B.B.); claudia.consales@enea.it (C.C.); 2 Department of Information and Communication Technologies, Pompeu Fabra University, 08002 Barcelona, Spain; tomas.garcia@upf.edu; 3 CNRS, Metabolic and Systemic Aspects of the Oncogenesis, (METSY), Institute Gustave Roussy, Paris-Saclay University, 94805 Villejuif, France; adeline.muscat@gustaveroussy.fr (A.M.); leslie.vallet@gustaveroussy.fr (L.V.); luis.mir@cnrs.fr (L.M.M.); franck.andre@cnrs.fr (F.A.)

**Keywords:** cell electrosensitization, fractionated electric pulse protocol, nanosecond pulsed electric fields, neuroblastoma cell, mesenchymal stem cell, calcium, electroporation

## Abstract

In recent years, the application of pulsed electric fields with very short durations (nanoseconds) and extremely high amplitudes (MV/m) has been investigated for novel medical purposes. Various electric protocols have been explored for different objectives, including the utilization of fractionated pulse doses to enhance cell electrosensitization to the uptake of different markers or an increase in apoptosis. This study focused on the use of fluorescence imaging to examine molecular calcium fluxes induced by different fractionated protocols of short electric pulses in neuroblastoma (SH-SY5Y) and mesenchymal stem cells (HaMSCs) that were electroporated using nanosecond pulsed electric fields. In our experimental setup, we did not observe cell electrosensitization in terms of an increase in calcium flux following the administration of fractionated doses of nanosecond pulsed electric fields with respect to the non-fractionated dose. However, we observed the targeted activation of calcium-dependent genes (*c-FOS*, *c-JUN*, *EGR1*, *NURR-1*, *β3-TUBULIN*) based on the duration of calcium flux, independent of the instantaneous levels achieved but solely dependent on the final plateau reached. This level of control may have potential applications in various medical and biological treatments that rely on calcium and the delivery of nanosecond pulsed electric fields.

## 1. Introduction

Cell electroporation followed by cell electropermeabilization [1] has gained widespread recognition for its ability to facilitate the uptake of various membrane-impermeable substances without inducing cell death. This technique has been exploited in clinical treatments such as electrochemotherapy or electro-gene transfer [2,3,4,5,6]. Conversely, pulsed electric fields with amplitudes higher than the ones used in electrochemotherapy have also been noted for their capacity to cause irreversible disruption to cell membranes and have been employed in treatments like oncologic irreversible electroporation or, more recently, in cardiac ablation techniques [7,8,9].

Given the pivotal role of electric pulse effects in numerous medical applications, extensive efforts are currently underway to investigate conditions that can amplify these effects, such as an increase in the uptake of drugs or other molecules and cell death, looking at the abovementioned clinical applications. This enhancement aims to improve the overall efficacy of electric pulse treatments [10,11].

**Figure 1:**
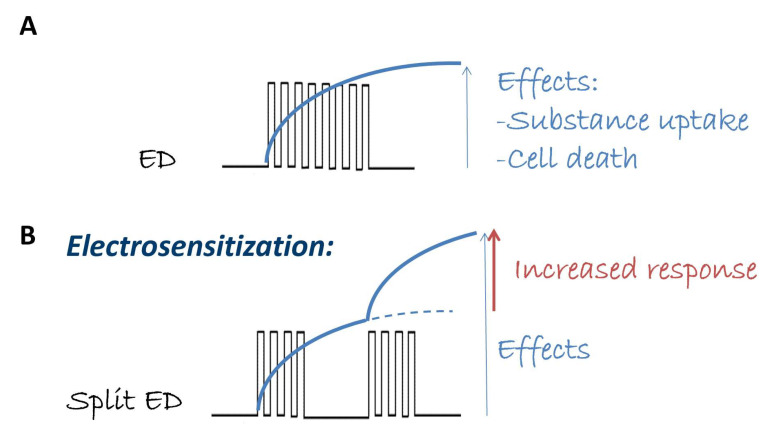
Schematic representation of electrosensitization. For a certain electric dose (ED) in terms of pulse number, amplitude, and repetition frequency that induces a specific effect (e.g., substance uptake, cell death, panel (**A**)), the electrosensitization effect occurs if the same ED in terms of pulse amplitude and repetition frequency, but delivered in multiple pulse fractions, is able to amplify the same effect (panel (**B**)).

In this context, the research group led by A. Pakhomov demonstrated that specific patterns of fractionated electric dosages enhance the desired effects of electric pulses when administered in vitro [10,11,12,13,14]. This phenomenon, referred to as “electrosensitization” and schematically described in Figure 1, involves splitting the electric dosage into multiple fractions during delivery. It is particularly intriguing due to its potential to reduce the required dosages in medical applications of pulsed electric fields and the possibility of a consequent reduction in the associated side effects. Specifically, the effect of pulsed electric fields increases significantly when a train of electric pulses is divided into nearly two fractions: the first fraction makes cells more sensitive to various effects (e.g., cell death, molecule uptake) caused by subsequent fractions, thereby making the entire treatment more efficient [14]. In fact, in many applications, the desired effect of electropermeabilization can only be achieved by delivering multiple pulses. In these cases, the first pulses already modify the target conditions and change the susceptibility to subsequent pulses (as illustrated in Figure 1). In [11], it is further emphasized that the waiting time between the two fractions is the critical parameter required to achieve a greater effect, where the pulse repetition frequency is not so important. This phenomenon could not be explained simply by membrane leakage and resealing, leading to the new concept of delayed electrosensitization caused by still unknown biological mechanisms, as indicated in [10,11,12,13,14].

In previous investigations, researchers have employed pulsed electric fields of varying durations, with some lasting hundreds of microseconds (µs) [12,13], while only the study by Pakhomova in 2011 [10] tested electric pulses of a few µs (4.5 and 9 µs). The authors explored electric pulses ranging from hundreds of nanoseconds (ns) down to 60 ns for electrosensitization in [10,11,14]. The most extensively investigated signals have mainly consisted of μs electric pulses or electric pulses lasting hundreds of ns. Therefore, it is worth noting that this phenomenon remains unexplored in the context of extremely short signals—for example, those lasting just a few ns (e.g., 10 ns).

Various endpoints have been examined to assess electrosensitization, including cell survival [10,12,14], electrosensitization for propidium iodide uptake (a well-known fluorescent dye used to detect cell membrane permeabilization) [10,11,12,13,14], and bleomycin uptake (a standard chemotherapeutic drug used in electrochemotherapy) [12]. Hence, the observations made so far have predominantly focused on cell survival and the uptake of different molecules. Furthermore, these observations have largely occurred post-pulse-application, rather than in real time.

In the current literature, electrosensitization has primarily been tested on transformed cells, such as U-937 cells (a human pro-monocytic model cell line), Jurkat cells (an immortalized T lymphocyte cell line), B16 murine melanoma cells, and KLN205 murine squamous cell carcinoma cells. Additionally, normal cells, such as Chinese Hamster Ovary (CHO) epithelial cell lines, have also been investigated [10,11,12,13,14]. Notably, this phenomenon has not yet been examined in cell cultures relevant to neuronal functionalities or in stem cells.

It is worth mentioning that in previous studies, in vitro exposure modalities have used standard electroporation cuvettes [10,11,12,14] as well as rod or needle electrodes to stimulate a specific number of cells [10,13,14].

In our study, our primary aim was to investigate the manifestation of the electrosensitization phenomenon across various signal patterns in the short ns time scale. We also split the electric dose into three fractions, which is a novel approach not explored before. Furthermore, we aimed to determine its applicability for a diverse biological endpoint that has not been explored, namely calcium (Ca^2+^) uptake.

In contrast to previous exposure modalities, in our study, we explored the electrosensitization effect by the following.

(i) Utilizing very short ns signals (10 ns) of high amplitude, up to 10 MV/m, and splitting the electric dose into up to three fractions to investigate the generality of the phenomenon for a fraction number higher than two.

(ii) Testing different cell types, including neuronal cancer cells like the neuroblastoma cell line SH-SY5Y [15], which could be useful in addressing neurological functions and disorders. Additionally, we applied our fractionated protocol on human mesenchymal stem cells (HaMSCs), which are of interest for potential use in regenerative medicine [16].

(iii) Assessing cell electrosensitization by observing Ca^2+^ fluxes in real time, i.e., recording the Ca^2+^ fluxes before, during, and after the delivery of the electric pulses in all the tested conditions. We used the fast FURA-2-AM Ca^2+^ indicator marker, and Ca^2+^ uptake represents an intriguing endpoint for medical applications in both cancer and regenerative medicine.

The electrosensitization of Ca^2+^ fluxes could be a significant endpoint as the controlled increase of Ca^2+^ is beneficial in regulating various cell functions, from differentiation to migration, which can be exploited in therapeutic applications. We monitored the fluorescence ratio of the emissions of FURA-2-AM excited at two different wavelengths (as detailed in Section 4) over time. The detection of electrosensitization relies on the achieved Ca^2+^ level, as depicted in Figure 1. If the phenomenon occurs, a higher level of Ca^2+^ uptake must be achieved when the electric protocol is delivered to cells in two or three fractions (Figure 1B).

In our experiments, we used a coplanar waveguide [17] to ensure a homogeneous electric field distribution on cell monolayers and the capability to deliver high electric field levels using thick gold electrodes (tens of micrometers). Our fractionated dose protocol was derived from the work of Jensen et al. [11], who conducted an extensive study to optimize the protocols for electrosensitization.

After the real-time imaging of Ca^2+^ fluxes, we assessed cell viability up to seven days post-exposure, as well as Ca^2+^-regulated genes responsible for various fundamental cell functions [18,19,20,21,22] at different time points post-exposure (up to 48 h). Our gene selection criteria involved genes known to be regulated in response to electrical stimulation, Ca^2+^ influx, or by the transcription factor CREB, which becomes activated through Ca^2+^-calmodulin protein kinases. This initial gene selection allowed us to conduct a preliminary assessment of our research objectives, observing direct downstream events related to the increase in Ca^2+^ uptake in the cells.

In our in vitro experiments, we did not observe cell electrosensitization in terms of an increase in Ca^2+^ fluxes following the administration of fractionated doses of ns pulsed electric fields lasting 10 ns. However, we observed the targeted activation of Ca^2+^-dependent genes related to synaptic plasticity and neurite outgrowth based on the total duration of Ca^2+^ fluxes, which increased when multiple fractions were administered to cells. This level of control may have potential applications in various medical and biological treatments reliant on the delivery of ns pulsed electric fields.

## 2. Results

### 2.1. Ca^2+^ Responses

**Figure 2:**
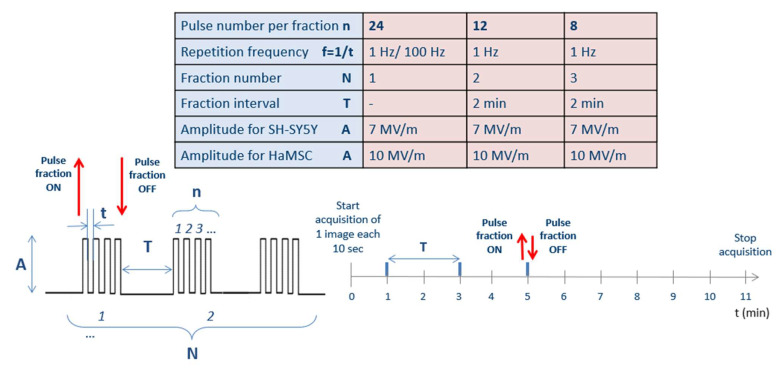
Delivered pulse protocols. Globally, always 24 pulses were delivered to cells, consecutively in an unique fraction (*N* = 1, with a repetition frequency f = 1/t of 1 Hz or 100 Hz) or arranged in multiple fractions (*N* = 2 or *N* = 3, with a 2-min period without pulses and with a pulse repetition frequency f = 1/t of 1 Hz), for a total of four different stimulation modalities for both cell lines. Fluorescence images were acquired every 10 s starting from 1 min before the exposure up to 10 min after the end of the exposure. Moreover, sham exposures were performed during each set of experiments. The experiments were repeated in triplicate.

**Figure 3:**
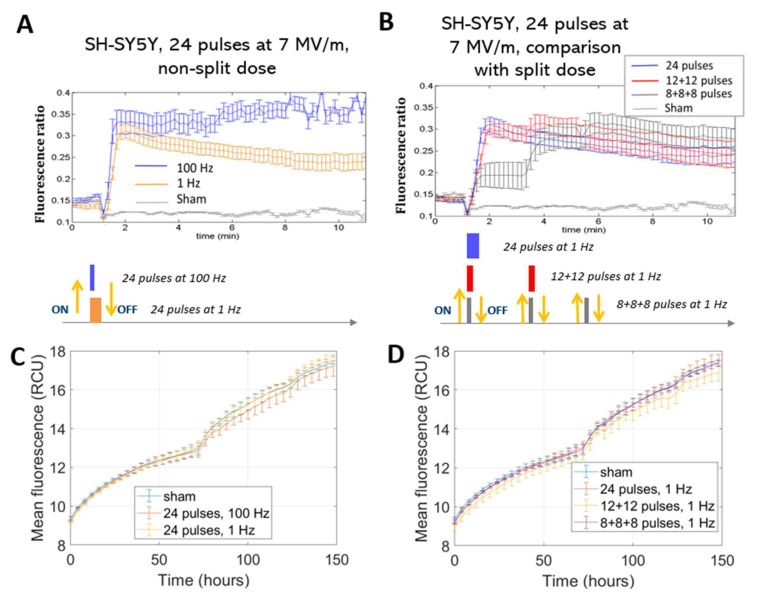
Ca^2+^ fluxes in SH-SY5Y cells (**A**,**B**). Fluorescence ratio of FURA-2-AM dye measured by time-lapse before and after pulse delivery using different exposure protocols: (**A**) 24 pulses at 1 Hz and 100 Hz and (**B**) 24 pulses at 1 Hz delivered consecutively or in multiple fractions (*p* < 0.05 in (**A**) starting from 3 min, in (**B**) during the first fraction of the 8 + 8 + 8 protocol). The diagram of the different pulse patterns applied is illustrated below the corresponding Ca^2+^ flow graph; each colored block represents a pulse fraction and is positioned at the time instant that it was applied. For ease of understanding, each block’s color matches the line in the above graph. Acquisition for 11 min, 1 image each 10 s. Viability of SH-SY5Y cells (**C**,**D**). Mean fluorescence of NucLight red dye measured at the end of exposure with (**C**) 24 pulses at 1 Hz and 100 Hz and (**D**) 24 pulses delivered consecutively or in multiple fractions at 1 Hz. Acquisition for 150 h, 1 image each 4 h. In all the graphs above, the different exposure conditions are compared to the ‘sham’ condition, which is the one with the absence of an electromagnetic field.

To study Ca^2+^ fluxes after ns electric field exposure, SH-SY5Y and HaMSC cells were treated with different protocols, as reported in Figure 2 (described in more detail in Section 4). Figure 3A shows results of the trend of Ca^2+^ flux for SH-SY5Y, comparing the two different tested repetition frequencies of 1 and 100 Hz. Statistically significantly (*p* < 0.05) higher Ca^2+^ fluxes are associated with a higher repetition frequency of the pulse trains. Figure 3B shows, instead, for the same cell type, the comparison between fluxes originating from different fractions of the same total number of electric pulses. We can see that at the end of the exposure, the final fluorescence in the experiments in which the electric pulses were delivered in different fractions reached the same level obtained by delivering the same number of pulses in a single burst. Therefore, no electrosensitization effect was detected, since we did not detect any amplification of the effect (Ca^2+^ influx) as per the phenomenon’s definition (see Section 1 and Figure 1). Panels C and D of the same figure show that SH-SY5Y cells exposed to all the protocols had the same proliferation rate for the non-exposed cells. In our experiments, it was not possible to identify the electrosensitization phenomenon for Ca^2+^ influxes into SH-SY5Y cells. Higher Ca^2+^ fluxes appeared to be associated with an increase in the repetition frequency of the pulse trains, ranging from 1 to 100 Hz (Figure 3A,B), rather than the splitting of the pulse number into multiple fractions.

This effect was observed in the stimulation patterns consisting of 24 pulses. 

**Figure 4:**
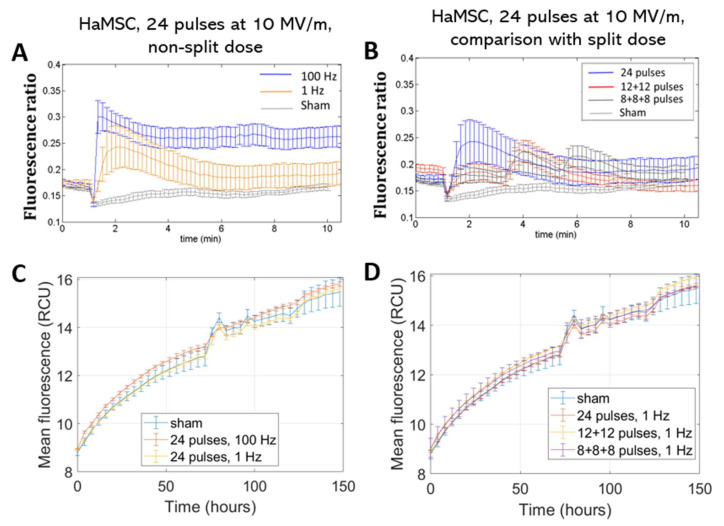
Ca^2+^ fluxes in HaMSC cells (**A**,**B**). Fluorescence ratio of FURA-2-AM dye measured by time-lapse before and after pulse delivery using different exposure protocols: (**A**) 24 pulses at 1 Hz and 100 Hz and (**B**) 24 pulses at 1 Hz delivered consecutively or in multiple fractions (*p* < 0.05 in (**A**) starting from 4 min). The stimulation patterns are the same as in Figure 2. Acquisition for 11 min, 1 image each 10 s. Proliferation of HaMSC cells (**C**,**D**). Mean fluorescence of NucLight red dye measured at the end of exposure with (**C**) 24 pulses at 1 Hz and 100 Hz and (**D**) 24 pulses delivered consecutively or in multiple fractions at 1 Hz. Acquisition for 150 h, 1 image each 4 h. ‘Sham’ condition indicates the absence of electromagnetic fields.

The same protocols were also administered to non-tumoral, non-excitable HaMSC stem cells. The same considerations can be made also for this cell line: we detected a greater effect for the higher repetition frequencies in a statistically significant manner (*p* < 0.05), but no electrosensitization was detected when splitting the electric dose into multiple fractions (Figure 4A,B). Cell viability, analyzed over time for HaMSCs (Figure 4C,D), similarly to the other cell type, did not show variations among the different tested electric protocols and the non-exposed samples. 

**Figure 5:**
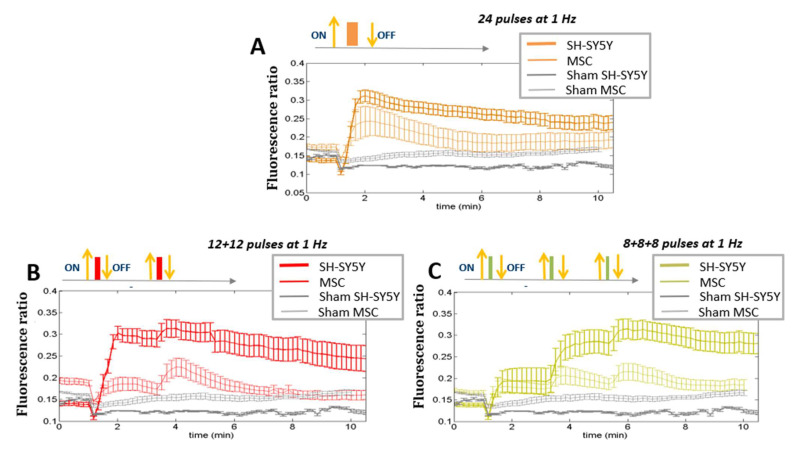
Ca^2+^ fluxes. Comparison among different protocols of pulses (24 pulses in one shot or in multiple fractions at 1 Hz) between SH-SY5Y (stimulated at 7 MV/m) and HaMSC cells (stimulated at 10 MV/m). ‘Sham’ indicates the absence of electromagnetic fields. (*p* < 0.05 starting from 1 min in panel (**A**,**B**), *p* < 0.05 from 3 min in panel (**C**)).

Furthermore, the lower Ca^2+^ fluxes of this cell type demonstrate the higher sensitivity of SH-SY5Y cells to electric pulse action. This fact is more evident when looking at Figure 5, where a direct comparison between the Ca^2+^ fluxes elicited in the two cell types is shown. HaMSC cells presented a higher threshold for electroporation and, even if they were stimulated with a more intense electric field, their Ca^2+^ fluxes were lower for all the tested protocols in a statistically significant manner (*p* < 0.05). The higher Ca^2+^ basal levels in HaMSC cells could be due to the high level of spontaneous Ca^2+^ waves of HaMSC cells [16]. 

### 2.2. Gene Expression Regulation

Due to the increased sensitivity to Ca^2+^ fluxes induced by pulsed electric fields observed in SH-SY5Y with respect to HaMSC cells, the expression of genes known to be regulated by Ca^2+^ fluxes were analyzed after electric pulse exposure by using different protocols. We studied the expression levels of the immediately early genes (IEGs), i.e., *Fos* proto-oncogene (*c-FOS*), *early growth response 1* (*EGR1*), and *Jun* proto-oncogene (*c-JUN*); the expression of the transcription factor *nuclear receptor subfamily 4 group A member 2* (*NURR-1*); the expression of two cytoskeletal proteins and markers of neurite outgrowth, *β3-TUBULIN* and *neurofilament light chain* (*NEFL*); and the expression of *brain-derived neurotrophic factor* (*BDNF*), involved in neuronal differentiation. 

**Figure 6:**
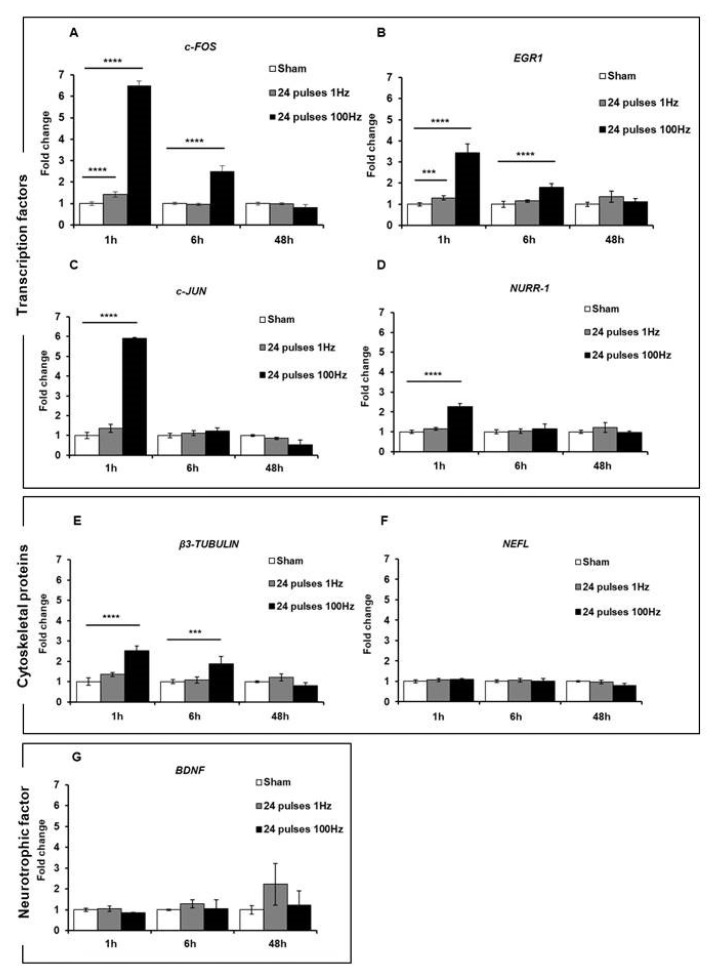
mRNA expression levels of transcription factors *c-FOS* (**A**), *EGR1* (**B**), *c-JUN* (**C**), and *NURR-1* (**D**); structural proteins *β3-TUBULIN* (**E**) and *NEFL* (**F**); and neurotrophic factor *BDNF* (**G**) were analyzed by qPCR after 1, 6, and 48 h of stimulation of SH-SY5Y cell line with 24 electric pulses at 1 Hz and 100 Hz. Data are reported as mean ± SD *** *p* < 0.001; **** *p* < 0.0001 for comparison with non-exposed groups (sham).

**Figure 7:**
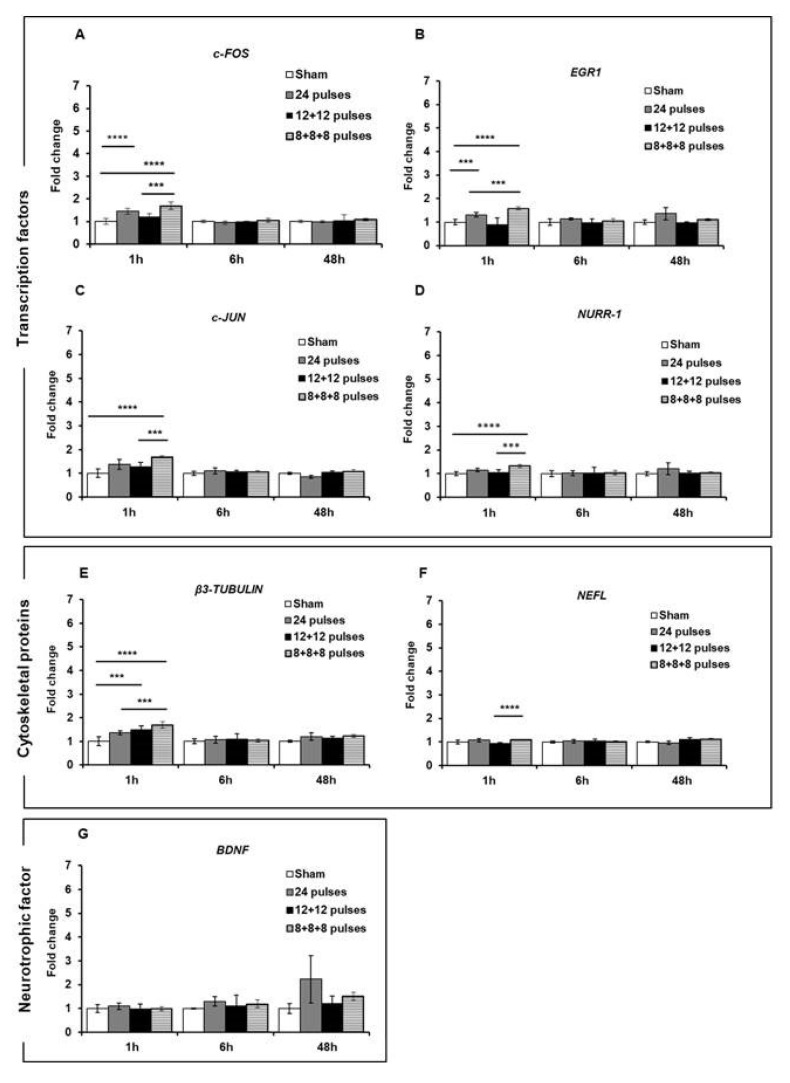
mRNA expression levels of transcription factors *c-FOS* (**A**), *EGR1* (**B**), *c-JUN* (**C**) and *NURR-1* (**D**); structural proteins *β3-TUBULIN* (**E**) and *NEFL* (**F**); and neurotrophic factor *BDNF* (**G**) were analyzed by qPCR after 1, 6, and 48 h of stimulation of SH-SY5Y cell line with 24 electric pulses at 1 Hz delivered consecutively or in multiple fractions. Data are reported as mean ± SD *** *p* < 0.001; **** *p* < 0.0001 for comparison with sham groups.

First of all, we assessed the expression of these genes in cells subjected to a 24-pulse treatment at the two repetition frequencies of 1 Hz and 100 Hz. As reported in Figure 6, 1 h after the exposure to 24 pulses at 1 Hz and 100 Hz, a statistically significant increase in the expression of *c-FOS* (1 Hz: *p* < 0.0001; 100 Hz *p* < 0.0001) and *EGR1* (1 Hz *p* = 0.0002; 100 Hz *p* < 0.0001) was observed at both frequencies compared to the sham-exposed cells, while the expression levels of the *c-JUN* (*p* < 0.0001), *NURR-1* (*p* < 0.0001), and *β3-TUBULIN* (*p* < 0.0001) genes were significantly augmented only after 100 Hz exposure. For the *BDNF* and *NEFL* genes, there was no increased expression after these treatment conditions. For all the genes, 6 h post-treatment, there was a gradual reduction in gene expression until their return to the basal levels of the sham-exposed group, although gene expression was still significant at 6 h for *c-FOS*, *EGR1*, and *β3-TUBULIN* (Figure 6). These results suggested that the Ca^2+^ fluxes, provoked by ns electrical stimulation, induced and increased the expression of some genes involved in synaptic plasticity and that this expression level was related to the repetition frequency of the pulse trains. To study the effects of fractionated pulse doses, we investigated the expression levels of the same genes analyzed above after cell exposure to 24 pulses, delivered consecutively or in multiple fractions (12 + 12 and 8 + 8 + 8) at only 1 Hz repetition frequency (Figure 7). As shown, the protocol 8 + 8 + 8 was more efficient in inducing an increase in the expression of *EGR1*, *c-JUN*, *NURR-1*, and *β3-TUBULIN* (*p* < 0.0001) compared to the protocol with pulses delivered consecutively, suggesting that fractionated doses seem to have a cumulative effect on gene expression related to the sustained Ca^2+^ fluxes during a longer time period. The levels of *BDNF* and *NEFL* remained unchanged after ns pulse exposure compared to the sham group, as well as with the split doses. This effect could have been due to the experimental settings, as explained in the Discussion.

## 3. Discussion 

In this paper, we investigated the phenomenon of electrosensitization induced by pulsed electric fields of high amplitude (MV/m) in the ns time range. Generally, pulsed-electric-field-based sensitization aims to enhance biological effects, such as cell death or the uptake of specific molecules, through the electroporation and subsequent electropermeabilization of cell membranes [1]. This enhancement is achieved by fractionating the total number of delivered electric pulses [10,11,12,13,14]. In this study, we employed either two or three fractions of electric pulses, with a total of 24 pulses. It is important to note that while Jensen et al. [11] determined that 24 pulses maximally amplified cell death, different pulse numbers might be required to induce electrosensitization for different endpoints compared to Jensen’s work [11], as in this study.

The findings based on the employed fractionated protocol suggest that there is no direct correlation, and therefore no electrosensitization, between the cell response in terms of Ca^2+^ fluxes (observed by the fluorescence ratio of the FURA-2-AM dye) and the applied stimulation with electric pulses when divided into multiple fractions. This lack of electrosensitization is specific to the current electric parameters and observation window used in the study. Notably, other research groups have observed cell electrosensitization for different endpoints with respect to the one here investigated (Ca^2+^ fluxes) using different types of electric pulses and other cell types [10,11,12,13,14]. These past observations included a decrease in cell survival and an increase in the uptake of various molecules, such as propidium iodide and bleomycin [10,11,12,13,14].

Furthermore, distinct responses were observed between SH-SY5H and HaMSC cells regarding Ca^2+^ fluxes and permeabilization levels, detected through the uptake of Ca^2+^ ions, as suggested in [16]. SH-SY5Y human neuronal cancer cells and HaMSCs both have relevance for therapeutic purposes in different medical areas, such as oncology and regenerative medicine, and, for this reason, they were used in our study. Moreover, both cell types are highly regulated by Ca^2+^. HaMSC cells lack the voltage-gated Ca^2+^ channels present in SH-SY5Y cells, which could contribute to this discrepancy. The membrane structure and composition can also influence these responses [4,23]. Further, the electroporation threshold can depend on the membrane composition in terms of the phospholipid type, as well as the cell size, although the latter remains a debated and controversial topic. For instance, HaMSC cells (with an average cell radius of 36 µm) require an electric field of 10 MV/m to observe Ca^2+^ uptake, while 7 MV/m suffices for SH-SY5Y cells (with an average cell radius of 12 µm) [15,16]. These amplitudes are used always for pulses lasting 10 ns considering all six presented protocols in the table of Figure 2. The occurrence of electroporation on both cell types can be directly evaluated by examining the Ca^2+^ fluxes (Figure 3, Figure 4 and Figure 5), as sham cells (not exposed to electric pulses) do not show modifications in their Ca^2+^ uptake over time. Finally, the different roles of Ca^2+^ in these cell lines could also explain the variations, particularly when considering the neural and excitable characteristics of SH-SY5Y cells.

Regarding cell viability, our tests conducted up to seven days post-exposure demonstrated that both cell lines remained viable and effectively restored membrane integrity after treatment. When we compared the viability of cells exposed to a single dose with those exposed to a fractionated dose, no differences were observed. This finding provides additional confirmation that there was no electrosensitization in both cell types under the studied conditions. In fact, a substantial increase in Ca^2+^ uptake (up to six-fold as predicted for electrosensitization in previous studies, [11]) would have decreased cell viability as high cytosolic Ca^2+^ levels are generally toxic for cells [24].

The analysis of Ca^2+^-regulated genes conducted in more Ca^2+^-sensitive cells (i.e., SH-SY5Y) revealed increased expression for some genes, even at 1 Hz, and, for all of these genes, higher expression at the highest repetition rate of 100 Hz. Ca^2+^-mediated signaling plays a crucial role in the generation of neuronal diversity, and brief elevations in intracellular Ca^2+^ levels, known as Ca^2+^ transients, are implicated in the regulation of various stages of neuronal development [25]. Genes known to be regulated by electrical stimulation on cells with electrical behavior, especially on neuronal and neuron-like cells, were analyzed [15,19,22,26,27]. Transcription factors *c-FOS*, *EGR1*, and *c-JUN* are immediate early genes (IEGs) that are activated rapidly and transiently and modulate synaptic plasticity and neurite outgrowth [19,28]. In accordance with other works where IEG expression was studied after electrical stimulation [15,26], our experimental conditions induced the increased expression of *c-FOS* and *EGR1* 1 h after 24 pulses at 1 Hz, while the expression of *c-JUN* increased at 100 Hz. The different response of *c-JUN* compared to *c-FOS*, which together form the AP-1 transcriptional complex, could be attributed to the analyzed experimental time. Carrasco and colleagues [26] observed a peak in expression at 15 min, returning to basal levels in 1 h. Meanwhile, for *c-FOS*, we observed increased expression at 1 h; for *c-JUN*, we might have already passed the activation window. The activation of *ERG1* and *c-FOS* was also observed in a previous study for a single electric pulse lasting 10 ns, with much lower intensity. This electric pulse was not able to permeabilize SH-SY5Y cells and allow Ca^2+^ flux entry from the outside. Therefore, the independent Ca^2+^ modulation of early gene activation cannot be completely ruled out [15]. The same trend as for *c-JUN* was observed for another transcription factor, *NURR-1*, which is activated during synaptic plasticity resulting from associative learning and has a CREB-binding site in its promoter region [19]. CREB is a Ca^2+^-responsive transcription factor activated by Ca^2+^-calmodulin protein kinases in neurons [29].

To further investigate synaptic plasticity, we also studied the expression of *β3-TUBULIN*, a marker of neurite outgrowth that has been reported to be regulated by Ca^2+^ under electrical and magnetic stimulation [22,30]. We also investigated *NEFL*, a cytoskeletal protein induced rapidly during synaptic plasticity and with a CREB-binding site in its promoter region [19], and the neurotrophic factor *BDNF* involved in neuronal differentiation and known to be regulated by electrical stimulation and Ca^2+^ influx [18,20,22]. While *β3-TUBULIN* was overexpressed at 100 Hz after stimulation, *NEFL* and *BDNF* did not change in our experimental settings. For *NEFL*, the explanation for its lack of change could be the analyzed experimental time, as previous research reported a peak in expression within 1 h [19]. For *BDNF*, the failure to induce its expression could have been due to a combination of the limited time window analyzed and technical considerations, as this gene has several transcript variants and our primers may not have amplified the correct one.

Importantly, for all the genes that showed a variation in their expression, even in the absence of electrosensitization for Ca^2+^ fluxes, we observed increased gene expression after stimulation with split doses. This suggests a cumulative effect possibly related to the longer duration for which Ca^2+^ remains above its basal threshold.

Therefore, it appears that the duration of increased Ca^2+^ flux levels determines the subsequent molecular cell response. In the electric dose fractionated into three groups, the final level of Ca^2+^ flux was the same as that obtained in the fractions of pulses divided into two and in the single fraction. The difference lay in the time delay at which the maximum Ca^2+^ fluxes were achieved. This observed dependence of the molecular response could potentially be used to control different cell functions. Notably, these genes are involved in several important cell processes, including differentiation, migration, and synaptic plasticity, hinting at possible applications in new therapies.

It is important to note that dose fractionation is also used in ionizing radiation delivery to make radiotherapy more effective [31]. Therefore, studying this phenomenon for non-ionizing radiation is of significant interest for therapeutic reasons. Furthermore, understanding the common molecular mechanisms activated by general stress factors, such as ionizing or non-ionizing radiation, which seems to mediate immediate responses by activating Ca^2+^ fluxes and downstream Ca^2+^ cell signaling, would be useful. Some studies have also suggested the involvement of reactive oxygen species (ROS) in the response to stress [4,23,31,32]. 

In this study, we also investigated possible pathways for Ca^2+^ entry, primarily from the extracellular environment. The endoplasmic reticulum and mitochondrial membranes did not appear to be affected by the short electric pulses used, as indicated by the absence of Ca^2+^ uptake when Ca^2+^ was absent in the extracellular medium and when a Ca^2+^ chelator (EGTA) was added to the cell solution (as shown in Supplementary Figure S1).

This study represents a first step, with some acknowledged limitations, in understanding the electrosensitization phenomenon of real-time Ca^2+^ flux and its molecular implications. Several aspects warrant further investigation, including observing Ca^2+^ fluxes at different and higher repetition frequencies and electric pulses durations, as well as analyzing various downstream molecular pathways over longer periods. In future research, we plan to focus on conducting a transcriptomic analysis to identify a broader array of genes that may be modulated. Additionally, the role of voltage-gated Ca^2+^ channels in Ca^2+^ uptake, potentially using channel blockers, should be explored to better understand the root of the phenomenon. Investigating the role of mitochondria in terms of membrane potential and ATP activation also presents an intriguing avenue for future research. Furthermore, exploring the long-term contributions of mitochondria and their role in reestablishing cell homeostasis after pulses requires further investigation. Finally, the modulation of ROS after splitting electric doses could also be another point of future research, given its potential role in cellular responses to stress [4,23,32].

## 4. Materials and Methods

### 4.1. Cell Culture

Human neuroblastoma cell line SH-SY5Y was obtained from the European Collection of Authenticated Cell Cultures (ECACC, Porton Down, Wiltshire, England). Cells were grown at 37 °C and 5% CO_2_ in Dulbecco’s modified Eagle’s medium/Ham’s F-12 (DMEM/F-12 50:50 mix), supplemented with 10% inactivated fetal bovine serum and 2 mM L-glutamine, 100 U/mL penicillin, and 100 µg/mL streptomycin and used before the fifteenth culture passage. 

Human adipose-derived mesenchymal stem cells, HaMSCs, were isolated from the surgical waste of individuals undergoing elective lipoaspiration. Samples were obtained after acquiring written informed consent from all the donors, in accordance with French and European legislation [33,34]. The lipoaspirates were surgical waste and, as such, the French legislation (Art.L. 1245-2 du Code de la Santé Publique) establishes that authorization from an ethics committee is not required. Cells were grown in Dulbecco’s modified Eagle’s medium (DMEM) supplemented with 10% fetal bovine serum, 100 U/mL penicillin, and 100 μg/mL streptomycin. Cell culture chemicals were purchased from Gibco (Life Technologies, Carlsbad, CA, USA). Cells were propagated at 37 °C in a humidified 5% CO_2_ atmosphere by passing them every 3–4 days (one passage corresponding to one doubling time of the population). HaMSCs were isolated and characterized as in [16].

### 4.2. Cell Exposure

**Figure 8:**
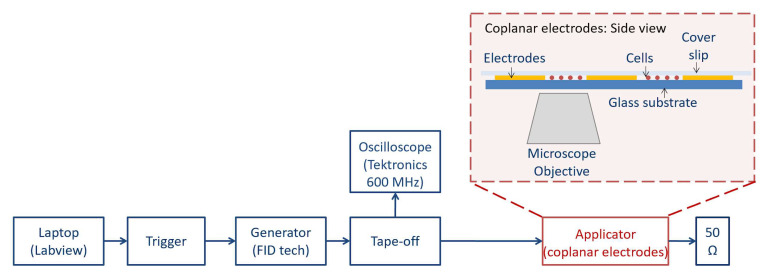
Block diagram of cell exposure system. The coplanar waveguide structure was integrated into an inverted microscope stage (Carl Zeiss, Jena, Germany). Electric pulses lasting 10 ns in different stimulation patterns were delivered by the FID high-voltage generator (FID GmbH, Burbach, Germany) triggered by the Agilent waveform generator 5386A (Agilent Technologies, Santa Clara, CA, USA) and controlled by an in-house LabVIEW 2016 program. The tape-off stage (BARTH® Elektronik GmbH, Lengerich, Germany) was necessary to send signals both to the coplanar electrodes and to an oscilloscope for monitoring (600 MHz TDS 3064B, Tektronix, Beaverton, OR, USA). An example of an acquired fluorescence image is reported in the Supplementary Materials (Figure S2).

To study Ca^2+^ fluxes, a coplanar waveguide structure was used. This exposure system was chosen for its wide frequency band, thus allowing us to test very short electric signals, in the ns time scale, in a very efficient way. The setup has been largely tested and its operational efficacy has been proven, as well as its biocompatibility with cells, in real-time experiments [17]. Electric field homogeneity was also optimal, with the electrode being much higher (tens of micrometers) compared to the single cell monolayer (few micrometers). In Figure 8, we provide the block diagram of the cell exposure system. The coplanar waveguide structure was integrated into an inverted microscope stage (Carl Zeiss, Jena, Germany). Electric pulses lasting 10 ns in different stimulation patterns were delivered by a FID Generator (FID GmbH, Burbach, Germany) triggered by an Agilent waveform generator 5386A (Agilent Technologies, Santa Clara, CA, USA) controlled by an in-house LabVIEW 2016 program. A tape-off stage (BARTH® Elektronik GmbH, Lengerich, Germany) was necessary to send a high-voltage signal both to the coplanar electrodes and to an oscilloscope for monitoring (600 MHz TDS 3064B, Tektronix, Beaverton, OR, USA). 

To study gene expression levels, an electroporation cuvette (1 mm gap) instead was used for the exposure, maintaining the same electric field level and homogeneity as in the coplanar waveguide due to its electrode height.

Globally, 24 pulses were delivered to cells as in [11], consecutively or arranged in multiple fractions (with 2 min of silencing between a fraction and another) for a total of four different stimulation modalities, including two different repetition frequencies (1 and 100 Hz). Figure 2 summarizes the delivered pulse protocols: there were different conditions that were explored for both cellular lines.

### 4.3. Ca^2+^ Flux Staining and Analysis

Cells were seeded at a density of 1.5 × 10^5^ and 6 × 10^4^ for SH-SY5Y and HaMSCs, respectively, on a cover slip treated with vitronectin (Gibco, Life Technologies, Carlsbad, CA, USA) to improve adhesion 24 h before the experiments.

Cells were stained using FURA-2-AM (Thermofisher, Waltham, MA, USA) and incubated in standard culture medium in the dark at 37 °C for 20 min. The analyzed Ca^2+^ fluxes due to pulse application were related to the uptake of the Ca^2+^ that was present in the culture medium. The FURA-2-AM ratiometric fluorescence dye was used as a marker of Ca^2+^ flux due to its sensitivity and to the possibility, after calibration, to correlate the fluorescence intensity to the effective Ca^2+^ concentration present in the cells after its uptake induced by pulses [35]. FURA-2-AM excitation was performed at two different wavelengths (340 nm and 380 nm), and the ratio of the emissions (acquired at 510 nm) at these wavelengths was utilized as an indicator of the amount of intracellular Ca^2+^. This ratio was crucial in eliminating common artifacts, such as variable dye concentrations, different illumination intensities and optical path lengths, background fluorescence noise, and artifacts stemming from the microscope’s acquisition hardware and software (ZEN Microscopy Software, blue edition 3.2). Furthermore, FURA-2-AM is cell-permeable and is a fast dye for Ca^2+^ acquisition, qualities that were of high interest in our experiments performing time-lapse monitoring on cells. 

Next, coverslips with attached cells were washed with PBS and positioned over the coplanar waveguide electrodes as reported in [35]. During exposure to electric pulses and time-lapse acquisitions, cells were immersed in a thin layer of culture medium, which contained Ca^2+^ (1.5 mM). We used a culture medium without phenol red to minimize the autofluorescent background during real-time acquisitions. Fluorescence images (in green) were acquired every 10 s starting from 1 min before the exposure up to 10 min after the end of the exposure, to monitor complete Ca^2+^ flux dynamics after exposure. Sham exposures were performed during each set of experiments. Each experiment was repeated in triplicate.

An example of an acquired fluorescence image is provided in the Supplementary Materials (Figure S2). The green intensity (Figure S2) was correlated to the effective Ca^2+^ concentration in the cells. Each experiment included a total of 68 images of the cells that had a fixed position in time but varied in their fluorescence intensity in time as a function of the Ca^2+^ flux.

Thus, for each experiment, cells were manually outlined on the first image of the series, and the intensity in this ROI was quantified in all 68 images and so followed in time; the fluxes obtained from each cell in an image were averaged and finally we obtained a further weighted average over at least 3 independent experiments performed at the same exposure conditions.

### 4.4. Cell Viability

Cell viability was analyzed using the IncuCyte Nuclight Rapid Red Cell Labeling Kit (Sartorius, Gottinga, Germany). Cells were seeded at a density of 1500 and 800 cells per well for SH-SY5Y and MSCs, respectively, in 96 multiwell plates after being exposed in the electroporation cuvette to 24 electric pulses, given consecutively or in multiple fractions at two different repetition frequencies (1 and 100 Hz). At the end of treatment, 0.4 µL in 100 µL of medium was added to each well containing cells. Cells were incubated for 30 min at room temperature and the plate was imaged in the IncuCyte S3 Live-Cell Analysis System (Sartorius, Gottinga, Germany). Images were acquired for 156 h, 1 image each 4 h. The Nuclight Rapid Red Cell was used to fluorescently label the nuclei of living cells without perturbing cell function or biology. 

### 4.5. Real-Time qPCR

Total RNA was isolated using the RNeasy Kit (QiaGenGmbH, Hilden, Germany), and 800 ng of total RNA was reverse-transcribed using the High-Capacity cDNA Reverse Transcription Kit (Thermo Fisher Scientific, Waltham, MA, USA). The qPCR amplifications were obtained with a StepOne Plus Real-Time PCR System (Thermo Fisher Scientific, Waltham, MA, USA) using the PowerUp SYBR Green master mix (Thermo Fisher Scientific, Waltham, MA, USA). The following primers (Metabion International AG, Planegg, Germany) were used: *GAPDH* fwd primer 5′-ATTCCACCCATGGCAAATTC-3′, rvs primer 5′-GGGATTTCCATTGATGACA-3′; *c-FOS* fwd primer 5′-TACTACCACTCACCCGCAGAC-3′, rvs primer 5′-GAATGAAGTTGGCACTGGAGAC-3′; *c-JUN* fwd primer 5′-CCAACTCATGCTAACGCAGC-3′, rvs primer 5′-TCTCTCCGTCGCAACTTGTC-3′, *EGR1* fwd primer 5′-CAGCAGCCTTCGCTAACC-3′, rvs primer 5′-CCACTGGGCAAGCGTAA-3′; *NURR-1* (*NR4A2*) fwd primer 5′-GCTGGACTCCCCATTGCTTT-3′, rvs primer 5′-TGGCTTCAGCCGAGTTACAG-3′; *β3-TUBULIN* fwd primer 5′-AGCGTCTACTACAACGAGGC-3′, rvs primer 5′-AAGAGATGTCCAAAGGCCCC-3′; *BDNF* fwd primer 5′-GATGCTGCAAACATGTCCATGAG-3′, rvs primer 5′-TTTTGTCTGCCGCCGTTACC-3′; *NEFL* fwd primer 5′-ACCAAGACCTCCTCAACGTG-3′, rvs primer 5′-AGCCACTGGTTATGCTTCCC-3′. The *GAPDH* expression level was used to normalize the mRNA expression of target genes. The quantity of mRNA relative to the reference gene was calculated by the 2−ΔCT method.

### 4.6. Statistics

Data were given as mean ± standard deviation (SD). All statistical analyses were carried out using the GraphPad Prism 6 software (GraphPad, San Diego, CA, USA). The Kolmogorov–Smirnov test was used to assess whether data were sampled from populations following a Gaussian distribution. Comparisons between groups were performed using the parametric *t*-test (significance taken as *p* < 0.05). All experiments were repeated three times.

## 5. Conclusions

In conclusion, in this work, we tested electrosensitization due to a pulsed electric field of 10 ns applied in multiple fractions on SH-SY5Y and HaMSC cells.

Our protocol did not show evidence of electrosensitization for Ca^2+^ fluxes analyzed in real-time using florescence images of FURA-2-AM in both cell lines.

SH-SY5Y cells, being more sensitive to Ca^2+^ fluxes, were also investigated for Ca^2+^-dependent IEGs, and the modulation of some genes was observed for the highest repetition frequency and the protocol split into three fractions of 8 + 8 + 8 electric pulses. This outcome demonstrated that the time duration over the basal level of Ca^2+^ flux was responsible for gene activation, rather than its intensity.

This can be useful to modulate cell function in different oncological and non-oncological therapies. Our study is the first step in understanding cell electrosensitization in real time, and future work looking at different electric protocols (electric pulse duration and repetition frequency) and molecular endpoints seems of interest in the field of electric-pulse-based applications in medicine and biology for the evaluation of new therapeutic opportunities.

## Data Availability

Data are available under request to the corresponding author.

## Supplementary Materials

The following supporting information can be downloaded at: https://www.mdpi.com/article/10.3390/ijms242115616/s1.
