# Supplementary material for: In Vitro Imaging and Molecular Characterization of Ca2+ Flux Modulation by Nanosecond Pulsed Electric Fields
Source: Int J Mol Sci. 2023 Oct 26;24(21):15616. doi: 10.3390/ijms242115616 (PMC10647260; doi:10.3390/ijms242115616)
Supplement: Supplementary file 1 [file ijms-24-15616-s001.zip › ijms-2624237-supplementary.pdf]

In Vitro Imaging and Molecular Characterization of Ca^{2+} Flux Modulation by Nanosecond Pulsed Electric Fields

Francesca Camera [1,†], Eleonora Colantoni [1,†], Tomas Garcia-Sanchez [2], Barbara Benassi [1], Claudia Consales [1], Adeline Muscat [3], Leslie Vallet [3], Luis M. Mir [3], Franck Andre [3] and Caterina Merla [1,*]

[1] Division of Health Protection Technologies, Italian National Agency for Energy, New Technologies and Sustainable Economic Development (ENEA), 00123 Rome, Italy; francesca.camera@enea.it (F.C.); eleonora.colantoni@enea.it (E.C.); barbara.benassi@enea.it (B.B.); claudia.consales@enea.it (C.C.)
[2] Department of Information and Communication Technologies, Universitat Pompeu Fabra, 08002 Barcellona, Spain; tomas.garcia@upf.edu
[3] CNRS, Metabolic and Systemic Aspects of the Oncogenesis, (METSY), Université Paris-Saclay, Institut Gustave Roussy, 94805 Villejuif, France; adeline.muscat@gustaveroussy.fr (A.M.); leslie.vallet@gustaveroussy.fr (L.V.); luis.mir@cnrs.fr (L.M.M.); franck.andre@cnrs.fr (F.A.)
* Correspondence: caterina.merla@enea.it; Tel.: +39-0630484616
† These authors equally contributed to the work.

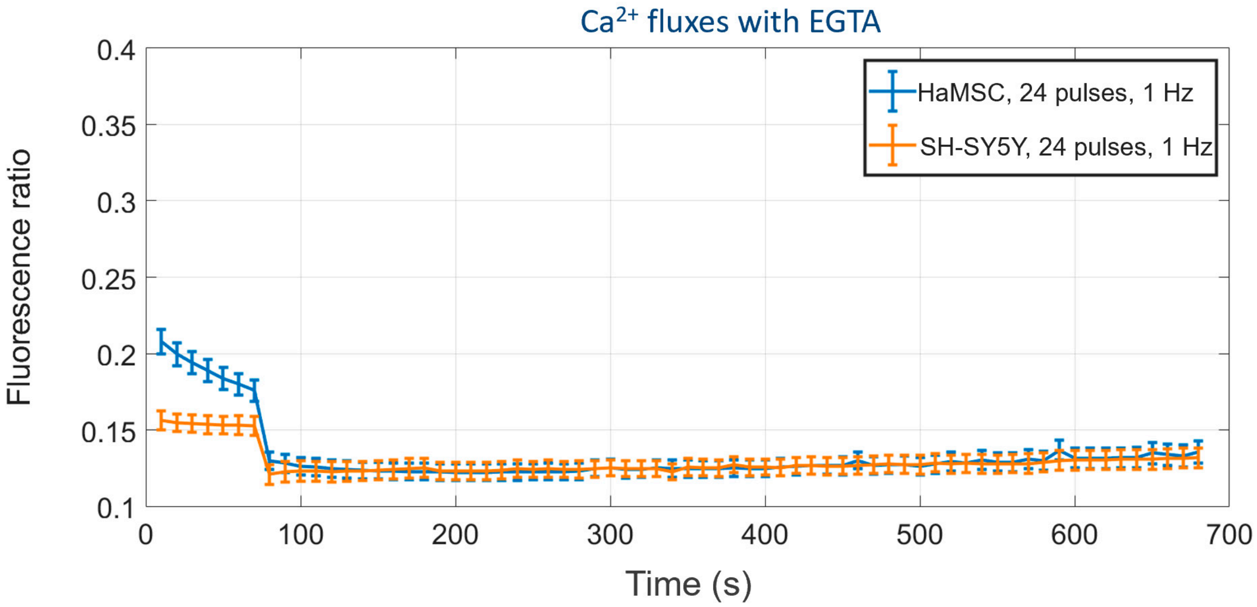

Figure S1. Ca^{2+} fluxes in HaMSC and SH-SY5Y cells in presefurnce of EGTA in the extracellular medium. Fluorescence ratio of FURA-2-AM dye measured by time-lapse before and after pulses delivery using for both cells the same protocol: 24 pulses at 1 Hz delivered consecutively ($p<0.05$). Acquisition for 11 minutes, 1 image each 10 seconds. The lack of calcium uptake when Ca^{2+} was absent in the extracellular medium indicates that mitochondrial membranes are not affected by the short electric pulses.

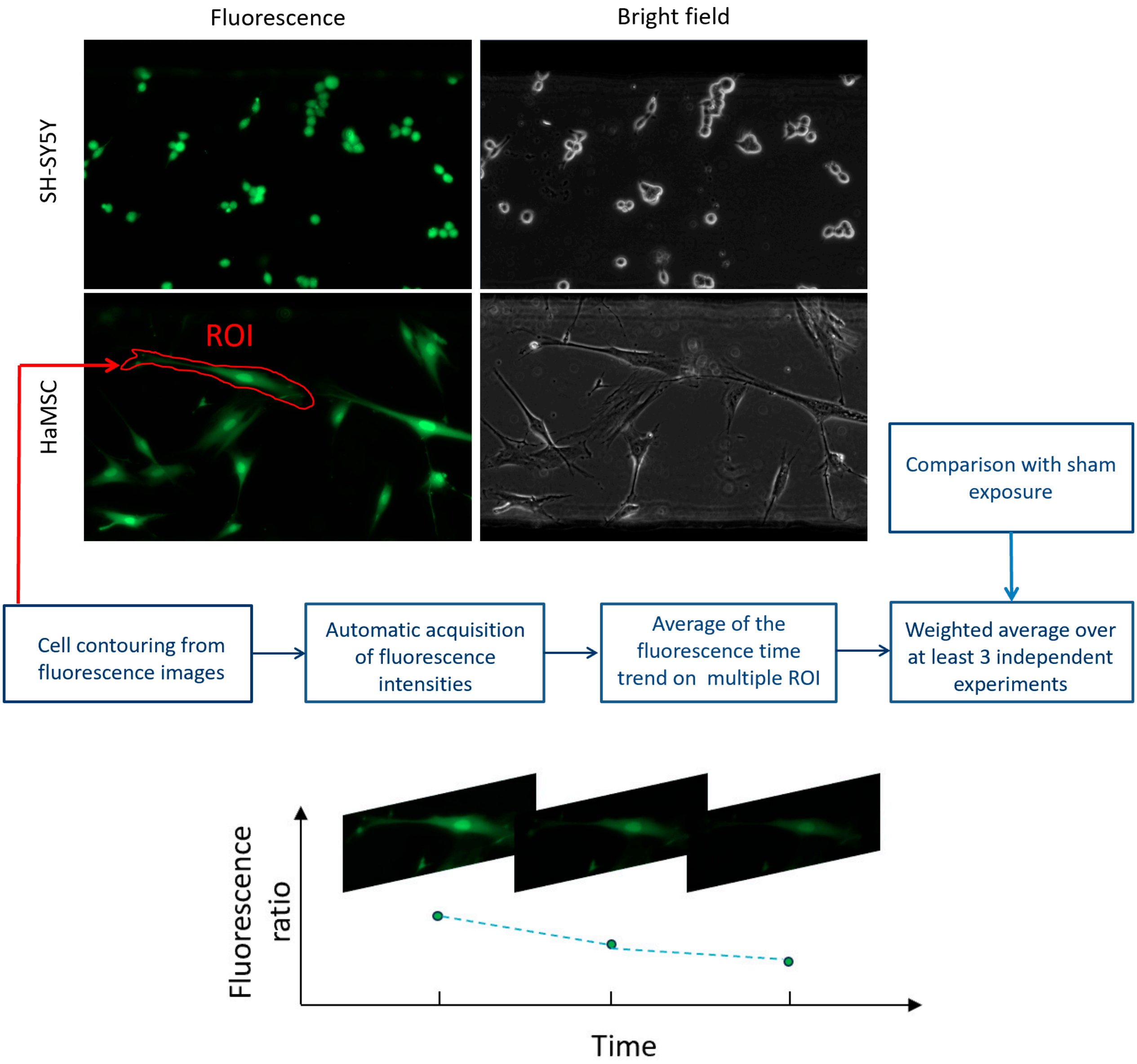

Figure S2. Block diagram of Ca^{2+} fluxes analysis and cell images (fluorescence and bright fields). This is an example of a fluorescence image acquired, in which the green intensity is correlated with the effective calcium concentrations present within the cells. In the images, the following steps were performed: 1) Cells were manually outlined to define a Region Of Interest (ROI); 2) The green intensity in that ROI was automatically acquired and tracked over time; 3) The fluxes obtained from each ROI were averaged, and 4) A further weighted average was calculated over at least 3 independent experiments performed under the same exposure conditions.
In the insert on the right, the time trend of the fluorescence ratio for a HaMSC cell is presented; these cells have an average radius dimension of around 36 μm. In the insert on the left, the time trend of the fluorescence ratio for a SH-SY5Y cell is shown; these cells have an average radius dimension of around 12 μm.
